# CRFR2 in paraventricular thalamus (PVT) mediates heat-induced anxiety in male mice

**DOI:** 10.1186/s13578-026-01624-w

**Published:** 2026-07-25

**Authors:** Ying Zhou, Shuqing Zhu, Ziyuan Ye, Shangwu Ye, Xiaomin Chen, Wenxi Chen, Jieshan Li, Gang Shu, Songbo Wang, Qingyan Jiang, Lina Wang

**Affiliations:** 1 Guangdong Provincial Key Laboratory of Animal Nutrition Control, South China Agricultural University, Guangzhou, 510642 Guangdong China; 2 National Engineering Research Center for Breeding Swine Industry, College of Animal Science, South China Agricultural University, Guangzhou, 510642 Guangdong China; 3 State Key Laboratory of Swine and Poultry Breeding Industry, Tianhe District, 483 Wushan Road, Guangzhou, 510642 Guangdong China; 4 Guangdong Laboratory for Lingnan Modern Agricultural, Guangzhou, 510642 Guangdong China

**Keywords:** Heat exposure, PVT, CRFR2, Anxiety-like behavior in the OFT

## Abstract

As global warming escalates, heat stress in both animals and humans has emerged as a pressing concern. As a physiological response to uncomfortable high ambient temperature, heat stress also significantly affects the emotional states of animals. The paraventricular thalamus (PVT), a pivotal brain region for stress response and emotion regulation, expresses many CRF receptors. However, the role of CRF receptors in the PVT in emotion regulation under heat conditions remains unclear. In our study, we revealed distinct sex-specific responses to heat conditions in mice: male mice presented significantly increased anxiety-like behavior, whereas female mice presented no significant alterations. After heat exposure, the male mice presented anxiety-like behavior, accompanied by a trend toward upregulation CRFR2 expression in the PVT and inhibition of the AKT/CREB signaling pathway. Conversely, reducing CRFR2 expression or inhibiting CRFR2 in the PVT effectively alleviated heat-induced anxiety-like behavior in the OFT. Furthermore, our findings revealed that chemogenetic activation of Vglut2 neurons in the PVT induced anxiety-like behavior primarily reflected in the OFT, whereas chemogenetic inhibition of these neurons mitigated heat-triggered anxiety in the OFT. Notably, the reduction in CRFR2 expression or the specific inhibition of CRFR2 in PVT^Vglut2^ neurons diminished heat-induced anxiety-like behavior in the OFT. Thus, our findings demonstrate that the CRFR2 and activity of Vglut2 neurons within the PVT contribute to the development of anxiety-like behavior in the OFT following heat exposure in male mice.

**Graphical abstract:**

**Figure:**
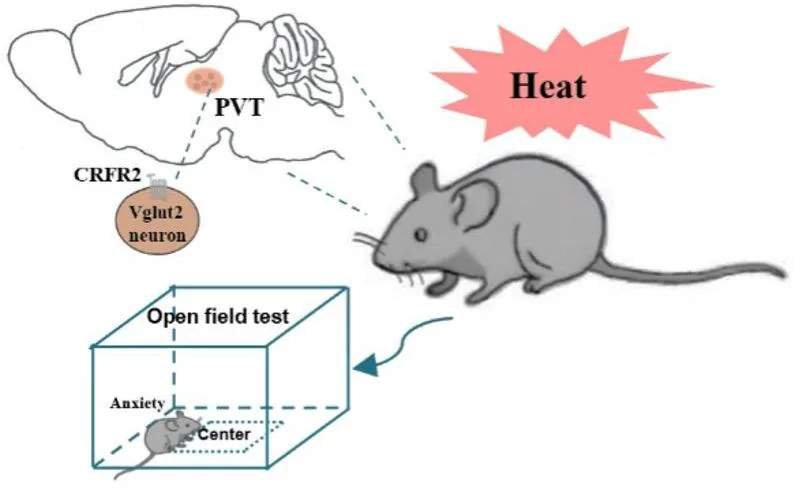

**Supplementary Information:**

The online version contains supplementary material available at https://doi.org/10.1186/s13578-026-01624-w.

## Introduction

With the advent of global warming, heat stress has emerged as a significant threat among the myriad of hazards posed by rising temperatures. It profoundly affects the physiological state of animals. Studies have shown that heat stress significantly affects an animal’s emotional state [1, 2]. Animals that experience heat stress often exhibit lethargy, rapid breathing, and anxiety‒depression symptoms, which may lead to fatal outcomes in extreme cases. Heat-exposed rats exhibit increased anxiety-like behavior and abnormal fear responses during elevated O-maze tests [2, 3]. Similarly, brief heat exposure can increase norepinephrine levels in the ventral hippocampus and dopamine levels in the ventral striatum of mice, whereas excessive core temperature may induce depressive-like behavior [4, 5]. Some studies have also shown that high temperatures affect primarily hippocampal structure and function, resulting in cognitive impairment and memory loss [6, 7].

The hypothalamic‒pituitary‒adrenal (HPA) axis serves as the principal stress‒response system in the body. This system involves the release of corticotropin-releasing hormone (CRH) from the paraventricular hypothalamic nucleus (PVH) and, subsequently, adrenocorticotropic hormone (ACTH), glucocorticoid (GC) and their feedback mechanisms. However, in addition to the classical PVH-mediated pathway, other brain regions also play a role in the stress response in animals. The paraventricular thalamus (PVT), a midline thalamic nucleus, serves as a crucial hub that integrates diverse behavioral pathways, including those related to emotion and reward-seeking behaviors [8–10]. The PVT integrates multiple input and output signals, helping the brain perceive and respond to environmental changes. For instance, glutamatergic neuron projections from the limbic prefrontal cortex to the PVT regulate motivation through cue-reward mechanisms [11]. Additionally, the PVT sends glutamatergic projections to several downstream brain regions—including the bed nucleus of the stria terminalis (BNST), periaqueductal gray (PAG), hypothalamus, NAc, and CeA—which regulate arousal, anxiety, and fear behaviors [12, 13]. Notably, PVT-NAc neurons are involved in processing cue–reward associations and aversive responses [14, 15] and play a role in depression following neural injury [16]. Different PVT neural circuits serve distinct physiological functions. PVT-suprachiasmatic nucleus (SCN) circuits control circadian rhythm [17], and PVT projections to the CeA regulate fear-related behavior [18].

Numerous studies have shown that CRF receptors are associated with emotional behaviors in animals [19–21]. Among them, through different brain regions and circuits, CRFR2 influences animal stress responses, emotions, and appetite, resulting in a variety of physiological and behavioral effects [22]. CRFR2-mutant mice exhibit increased anxiety-like behavior [23]. On the other hand, research has shown that CRFR2 is involved in regulating sex differences in social behavior [24]. These findings suggest that CRFR2 may function differently in stress responses depending on sex. Studies have shown that male mice with CRFR2 mutations exhibit anxiety-like behavior [25], and variations in CRFR2 expression in the DRN contribute to sex differences in emotional behavior [26]. Our previous study further confirmed that CRFR2 in the PVT modulates feeding behavior in male mice under cold stress but has no effect on female mice [27]. However, whether CRFR2 in the PVT participates in emotional behavior regulation under heat conditions remains elusive. Therefore, after the mice were exposed to acute heat, anxiety-like behavior, neuronal activity, and CRFR2 expression in the PVT of the mice were tested. The activation, inhibition and knockdown of CRFR2 in the PVT and the activation and inhibition of CRFR2-expressing neurons were subsequently carried out to verify the role of CRFR2 in the regulation of heat-induced anxiety-like behavior in mice.

## Materials and methods

### Animals

Eight-week-old C57BL/6J wild-type mice were procured from Zhuhai BesTest (Guangdong, China), and age-matched Rosa26-CAG-LSL-Cas9-tdTomato mice were obtained from GemPharmatech (T002249, Guangzhou, China). All the mice were group-housed (4–5 per cage) in a temperature- and humidity-controlled room maintained at 25 °C ± 1 °C and 60% relative humidity with a 12-h light/dark cycle (lights on at 06:30) and *ad libitum* access to food and water. Given the prolonged duration of heat exposure and the experimental protocol designed for a single sex, heat exposure experiments for male and female mice were performed on two separate days. To minimize errors associated with circadian rhythm variations among mice, both male and female mice were tested at the same time point each day. Behavioral assessments were conducted between 10:00 and 18:00 during the light phase to ensure circadian consistency.

### Heat exposure protocol

All the mice were group-housed (4–5 per cage) in a temperature- and humidity-controlled room, with strictly balanced grouping, age, body weight, and housing conditions. Notably, the mice did not undergo either heat exposure or control treatment in a crossover manner. A climate chamber was used to achieve the heat exposure condition, and the duration of exposure of the mice to 37 °C was 2 h. All the mice were then transferred to the anxiety behavioral tests immediately after heat exposure.

## Stereotaxic surgery, virus injection and cannula implantation

For stereotaxic surgery, the mice were anesthetized with 4–5% isoflurane (R510-22-10; RWD, China). Their eyes were coated with erythromycin eye ointment to prevent light blindness. The scalp was cut along the midline to expose the skull, and the mucosa on the skull surface was cleaned with a cotton swab. The skull was adjusted to ensure that it was level. After small holes in the skull were drilled, the virus was injected bilaterally or ipsilaterally into the PVT using a glass pipette mounted on a stereotaxic frame (bregma coordinates: antero-posterior (AP) = -0.46 mm; mediolateral (ML) = 0.00 mm; dorsal-ventral (DV) = -3.80 mm from the brain surface). The virus volume ranged from 200 to 300 nL per side and was infused at 100 nL/min. The needle remained in place for 3 min per 100 nL, and the injection pipette was withdrawn 10 min after the completion of the infusion. After surgery, the mice were allowed to recover from anesthesia on a 37 °C heating pad.

For cannula implantation, the mice were anesthetized with isoflurane and placed in an ultraprecise mouse stereotaxic frame. A guide cannula (62003, RWD, China) was implanted bilaterally over the PVT (-0.46 mm AP; 0.00 mm ML; -3.70 mm DV) of C57BL/6J mice that were a priori validated. The cannula was anchored to the skull surface with dental cement. Following implantation, a dummy cannula (62102, RWD, China) with a 0.5 mm extension beyond the guide cannula end and a metal cap was inserted into the guide cannula. The mice recovered for at least 7 days before testing.

### Cannula infusion in the PVT

One week after cannula implantation surgery, 0.5 µL of 100 µM or 200 µM CRFR2 agonist Urocortin III (UCN3, HY-P1858A, MCE, China) or 100 µM CRFR2 antagonist Astressin 2B (HY-P1108, MCE, China) was injected into the PVT [28–30]. For reagent infusion, UCN3 or Astressin 2B was dissolved in saline according to the manufacturer’s instructions. The reagent (0.5 µL) was then infused into the PVT through the injector cannula (62203; RWD, China). The injector cannula remained in the PVT for an additional 2–3 min to ensure adequate local reagent diffusion and minimize spread along the injection track. At ambient temperature (25 °C), the mice received a microinjection of saline, UCN3 or Astressin 2B, followed by OFT or EPM testing or perfusion for brain tissue collection 30 min post-injection. For the heat exposure test, the mice were microinjected with saline or Astressin 2B prior to heat exposure at 37 °C. After a 2 h period of heat exposure, the animals were subjected to either OFT/EPM behavioral testing or perfusion for brain tissue collection. After all the procedures were completed, fluorescence imaging was performed to verify the infusion sites. Data from PVT-cannulated mice with incorrect positions were excluded from the analysis (Fig. S2A–S2B).

### Knockdown of CRFR2 in the PVT of mice

Cas9 expression was initiated in Cre-dependent Cas9-expressing transgenic mice by injecting AAV2/9-hysn-Cre-WPRE-PA virus (Brain VTA, China) into the PVT region. This was followed by injection of either AAV2/9-H1-sgRNA(NC)sp-CAG-DIO-mCherry-WPRE-pA or AAV2/9-(H1-sgRNA(CRFR2)x3)-CAG-DIO-mCherry-WPRE-SV40pA (Taitool, China) to mediate CRFR2 knockdown in the PVT. The mice were housed in cages with adequate food and water. Behavioral tests were conducted approximately 3–4 weeks after virus injection. After all the behavioral procedures were completed, fluorescence imaging was performed to verify the accuracy of the sites. Data from mice with inaccurate positioned injections were excluded from the analysis (Fig. S2C). The number of CRFR2-positive neurons was quantified by immunofluorescence staining combined with ImageJ analysis.

### CRFR2 knockdown on Vglut2 neurons in the PVT

The rAAV-CMV-DIO-SaCas9-WPREs (Brain VTA, China) were injected into the PVT region of Vglut2-Cre mice. This was followed by the injection of either AAV2/9-H1-sgRNA(NC)sp-CAG-DIO-mCherry-WPRE-pA or AAV2/9-(H1-sgRNA(CRFR2)x3)-CAG-DIO-mCherry-WPRE-SV40pA to achieve CRFR2 knockdown on Vglut2 neurons in the PVT. The mice were housed in cages with adequate food and water. Behavioral tests were conducted approximately 3–4 weeks after virus injection. After all the behavioral procedures were completed, fluorescence imaging was performed to verify the accuracy of the sites. Data from mice with inaccurate positioned injections were excluded from the analysis (Fig. S2D).

### Chemo-genetic manipulation

For the chemo-genetic activation or inhibition experiments, the mice were habituated to the handling of the experimenter for at least 3 days. To activate or inhibit Vglut2 neurons in the PVT, each C57BL/6J mouse was first injected with rAAV9-VGLUT2-CRE-WPRE-hGH-polyA (Brain VTA, China) in the PVT, followed by AAV9-hSyn-DIO-hM3D (Gq)-mCherry or AAV9-hSyn-DIO-hM4D (Gi)-mCherry (Brain VTA, China). After virus injection surgery and three weeks of recovery, behavioral tests started. To modulate the function of Vglut2 neurons in the PVT of mice, saline or CNO (1 mg/kg, C0832; Sigma‒Aldrich, USA) was injected intraperitoneally. For the chemo-genetic activation or inhibition experiments at ambient temperature (25 °C), the animals received an independent injection of saline or CNO 30 min before OFT/EPM/perfusion for brain tissue collection, and no repeated administration was performed on the same day across the different paradigms. For the chemo-genetic inhibition experiments involving heat exposure (37 °C), the mice were microinjected with saline or CNO prior to heat exposure at 37 °C. After a 2 h period of heat exposure, the animals were subjected to either OFT/EPM behavioral testing or perfusion for brain tissue collection. After all the procedures were completed, fluorescence imaging was performed to verify the accuracy of the sites. Mice with incorrectly targeted viruses or tracers were excluded from the data analysis (Fig. S2E–S2F).

### Open field test (OFT)

To assess anxiety-like behavior, the mice were subjected to the OFT as described previously [31]. During behavioral testing, each mouse was placed in the center of a 50 cm × 50 cm × 30 cm arena and allowed to explore freely for 5 min under dim light (10–15 lux). A video camera mounted directly above the arena was used to record locomotor activity, which was analyzed using SuperMaze software (Xinruan Inc., China). An entry was counted only when all four paws of the mouse completely entered the target area. If only the head or front paws extended into the arm without full entry, it was not counted as an entry. The time spent in the center, distance traveled, number of central entries, and percentage of distance in the center were quantified as indicators of anxiety-like behavior. The apparatus was thoroughly cleaned between trials: all surfaces were wiped with 75% ethanol, followed by a 5-minute odor-neutralization interval before the next trial.

### Elevated plus-maze (EPM)

The EPM test was carried out as previously reported [32, 33]. The mice were placed in the EPM apparatus, which consisted of a central junction (9 cm by 9 cm), and four arms elevated 35 cm above the floor, with each arm positioned 90° relative to the adjacent arms. The two closed arms included high walls (35 cm by 9 cm by 20 cm), and the open arms were exposed (35 cm by 9 cm). Each mouse was positioned at the center of the maze with its head facing one of the open arms and was given 5 min to freely explore under light delivery. Total entries, time, and distance into the open, center, and closed arms were measured by SuperMaze software. For the EPM test, a valid entry of a mouse into a single arm was defined as one entry only when all four paws of the mouse completely entered the interior of the arm. Partial entry, including only head poking or forepaw extension into the arm without full entry of all four paws, was not counted as a valid entry. The following behavioral parameters were recorded and analyzed: the number of entries into the open arms, the total time spent in the open arms, the total distance traveled during the test, the percentage of open arm entries (calculated as the ratio of open arm entries to the total entries), and the number of entries into the closed arms. Between trials, the apparatus was sanitized by wiping all surfaces with 75% ethanol and allowing a 5-minute odor-neutralization period before subsequent testing.

OFTs and EPMs were conducted on separate days at intervals of ≥ 3 days to reduce cumulative stress in the mice.

### Western blotting

The mice were euthanized under isoflurane anesthesia, and their brains were rapidly removed and frozen on dry ice. The PVT and PVH regions were then microdissected using punches for further study. Total protein was extracted from punched brain tissue from the PVT and PVH using RIPA lysis buffer supplemented with 1 mM PMSF, and the protein concentration was measured using a BCA protein assay kit (89901; Thermo Fisher Scientific, USA). After separation on 10% sodium dodecyl sulfate‒polyacrylamide gel electrophoresis (SDS‒PAGE) gels, the proteins were transferred to polyvinylidene fluoride (PVDF) membranes and blocked with 6% (wt/vol) nonfat dry milk for 2 h at room temperature. The PVDF membranes were subjected to immunoblotting with primary antibodies against CRFR2 (1:1000; LS-B9098; Lifespan, USA), p-AKT (1:1000; 4060 S; CST, China), AKT (1:2000; 4691 S; CST, China), p-CREB (1:1000; 9198 S; CST, China), CREB (1:1000; 9197 S; CST, China), and tubulin (1:1000; sc-166729; Santa Cruz Biotechnology, China) at 4 °C overnight. The membranes were then incubated with a goat anti-rabbit HRP-conjugated secondary antibody (1:50000; BS13278; Bioworld, USA) for 1 h at room temperature. Target bands were detected using a fluorescence imaging system (Tanon 5200, Tanon, China) and quantified using ImageJ software. Western blotting of total AKT and total CREB was performed with blotted membranes stripped from the corresponding phosphorylated proteins, and the CRFR2 protein levels were normalized to the tubulin levels in each sample.

### Immunofluorescence staining

The mice were subsequently anesthetized with isoflurane and perfused with saline and 4% paraformaldehyde (PFA). The brains were obtained and postfixed overnight in 4% PFA and then dehydrated in 30% sucrose. The brains were cut into approximately 30 μm-thick coronal brain slices via a freezing microtome, and the brain slices were subsequently washed in PBS containing 0.1% Triton X-100 (PBS-T, pH 7.4) for 10 min each. The brain slices were then blocked in PBST containing 5% goat serum for 2 h. Primary antibodies against CRFR2 (1:750; LS-B9098; Lifespan, USA), CaMKIIα (1:500; 50049 S; CST, China), or c-Fos (1:1000; 2250 S; CST, China) were added to the slices, which were then incubated on an oscillator overnight at 4 °C. The slices were washed three times in 0.1% PBST the next day, with a ten-minute interval between each wash. On the second day, the brain slices were incubated with secondary goat anti-rabbit Cy3 (1:1000, 111-165-045; Jackson ImmunoResearch) or goat anti-rabbit 488 (1:1000, 111-545-003; Jackson ImmunoResearch) or goat anti-mouse 488 (1:1000, 115-545-003; Jackson ImmunoResearch) antibodies at room temperature for approximately 2 h in the dark and then sealed, after which the fluorescence intensity was visualized, and images were acquired via a Nikon imaging system (Nikon Ti-U738270; Nikon, China). For CRFR2 and CaMKIIa immunofluorescence staining, mouse brain slices were attached to adhesive glass slides and dried for 1 h, followed by thermal repair with sodium citrate repair solution (P0081; Beyotime). ImageJ was used to count the number of positive neurons.

### Statistical analysis

All the data are presented as the means ± standard errors of the means (S.E.M.). Statistical analysis was performed via GraphPad Prism 9.0. Differences between the control and treated groups were analyzed by Student’s *t* test. A significance (alpha) level of *P* < 0.05 was considered to indicate statistical significance.

## Results

### Heat-induced anxiety-like behavior in male mice and CRFR2 signaling in the PVT were influenced

**Fig. 1:**
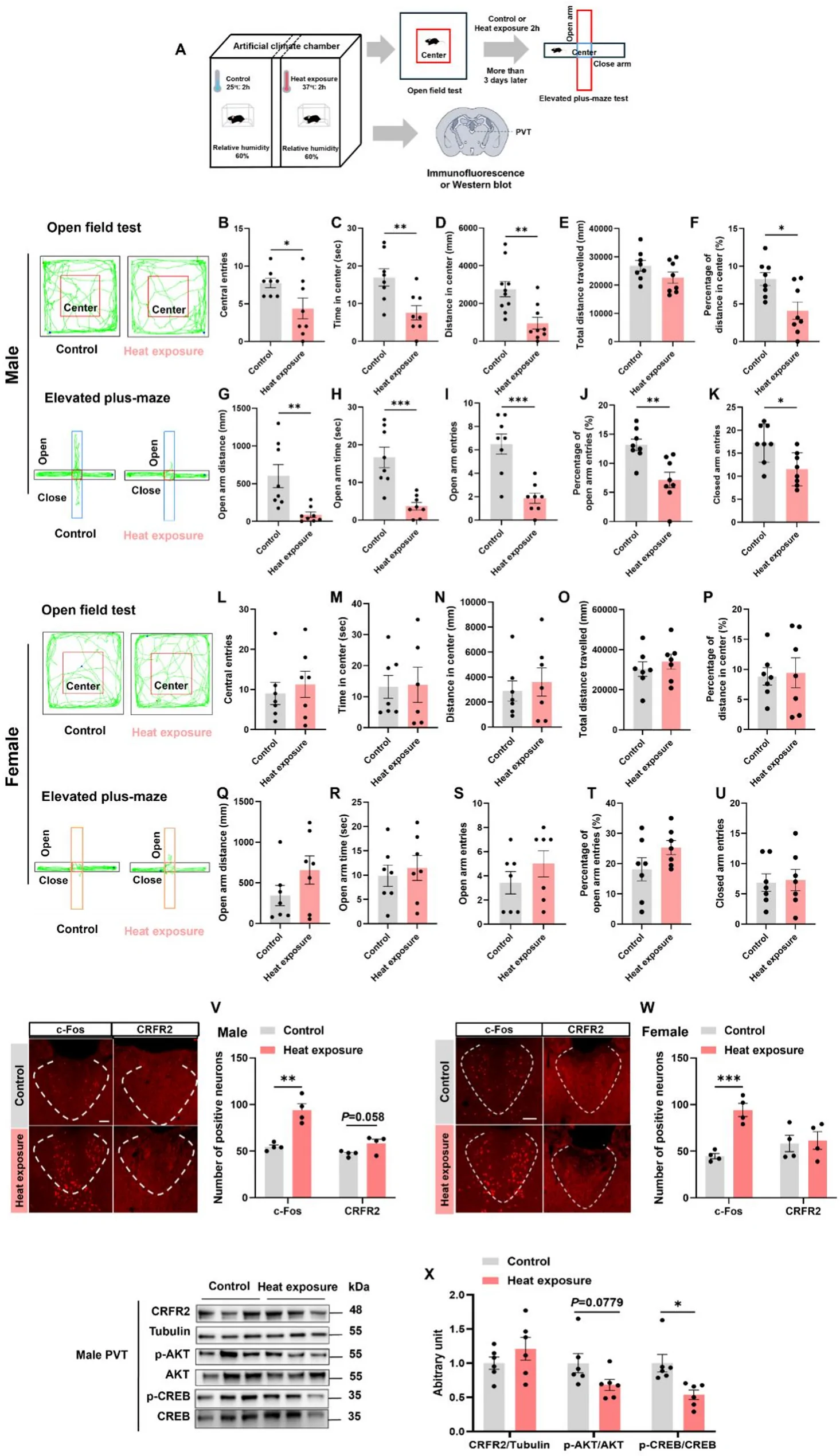
Heat-exposed male mice presented anxiety-like behavior. **A** Schematic diagram of heat exposure and timeline of the anxiety-like behavior test and immunofluorescence staining test (8-week-old male and female mice). **B**–**F** Open field test: central entries, time in the center, distance traveled in the center, total distance traveled, and percentage of distance traveled in the center (%) (males, *n* = 8 per group). Representative trajectory plots depict the behavioral patterns of a single representative subject. **G**–**K** Elevated plus-maze test: open arm distance, open arm time, number of open arm entries, percentage of open arm entries, and number of closed arm entries (male mice, *n* = 7 per group). Representative trajectory plots represent the behavior of a single representative subject. **L**‒**P** Open field test: central entries, time in the center, distance traveled in the center, total distance traveled, and percentage of distance traveled in the center (%) (female mice, *n* = 7 per group). Representative trajectory plots depict the behavioral patterns of a single representative subject. **Q**–**U** Elevated plus-maze test: open arm distance, open arm time, number of open arm entries, percentage of open arm entries, and number of closed arm entries (female mice, *n* = 7 per group). Representative trajectory plots represent the behavior of a single representative subject. **V**‒**W** c-Fos and CRFR2 expression in the PVT (in male and female mice; *n* = 4 per group). Scale bars, 200 μm. **X** CRFR2 protein, phosphorylated AKT and CREB levels in the PVT (in male mice, *n* = 6 per group). Data are presented as the mean ± SEM, and *P* values were determined using unpaired *t* tests (**B**–**U**) or multiple unpaired *t* tests (**V**–**X**). *P < 0.05, ** *P* < 0.01, ***P < 0.001

To clarify the effects of heat on mice, a control group and a heat exposure group were established. Both male and female mice were exposed to 37 °C for 2 h. Comprehensive behavioral analysis and immunofluorescence staining were performed to explore the potential connection between CRFR2 in the PVT and heat-induced anxiety (Fig. 1A). In the open field test (OFT), the number of entries (Fig. 1B), time spent (Fig. 1C), distance traveled in the center area (Fig. 1D), and the percentage of the distance traveled by the mice in the center area (Fig. 1F) was reduced, which is suggestive of anxiety. Similarly, in the elevated plus-maze (EPM), these mice also exhibited increased anxiety-like behavior, as evidenced by their decreased movement distance (Fig. 1G), reduced time spent (Fig. 1H), and fewer entries (Fig. 1I) and percentage of open arm entries (Fig. 1J) into the open arms. Interestingly, while heat-exposed male mice exhibited significant anxiety-like behavior, female mice did not exhibit such a phenotype (Fig. 1L and U). Moreover, a significant increase in c-Fos expression in the PVT was detected in heat-exposed male and female mice (Fig. 1V and W). In male mice, heat exposure led to an increasing trend in CRFR2 levels in the PVT (*P* = 0.058), while no such trend was observed in females (Fig. 1V and W). In addition, heat exposure suppresses AKT/CREB signaling in the PVT of male mice (Fig. 1X), which may increase the functional contribution of CRFR2 to heat-related behavioral changes [27]. However, in the PVH of mice, only the c-Fos level increased after heat exposure (Fig. S1A), with no significant effect on the expression of CRFR2 or its downstream signaling pathway (Fig. S1B). These results suggested that the CRFR2 signaling pathway in the PVT may participate in the regulation of heat-induced anxiety-like behavior in male mice.

### Local PVT infusion of CRFR2 agonists induced anxiety-like behavior

**Fig. 2:**
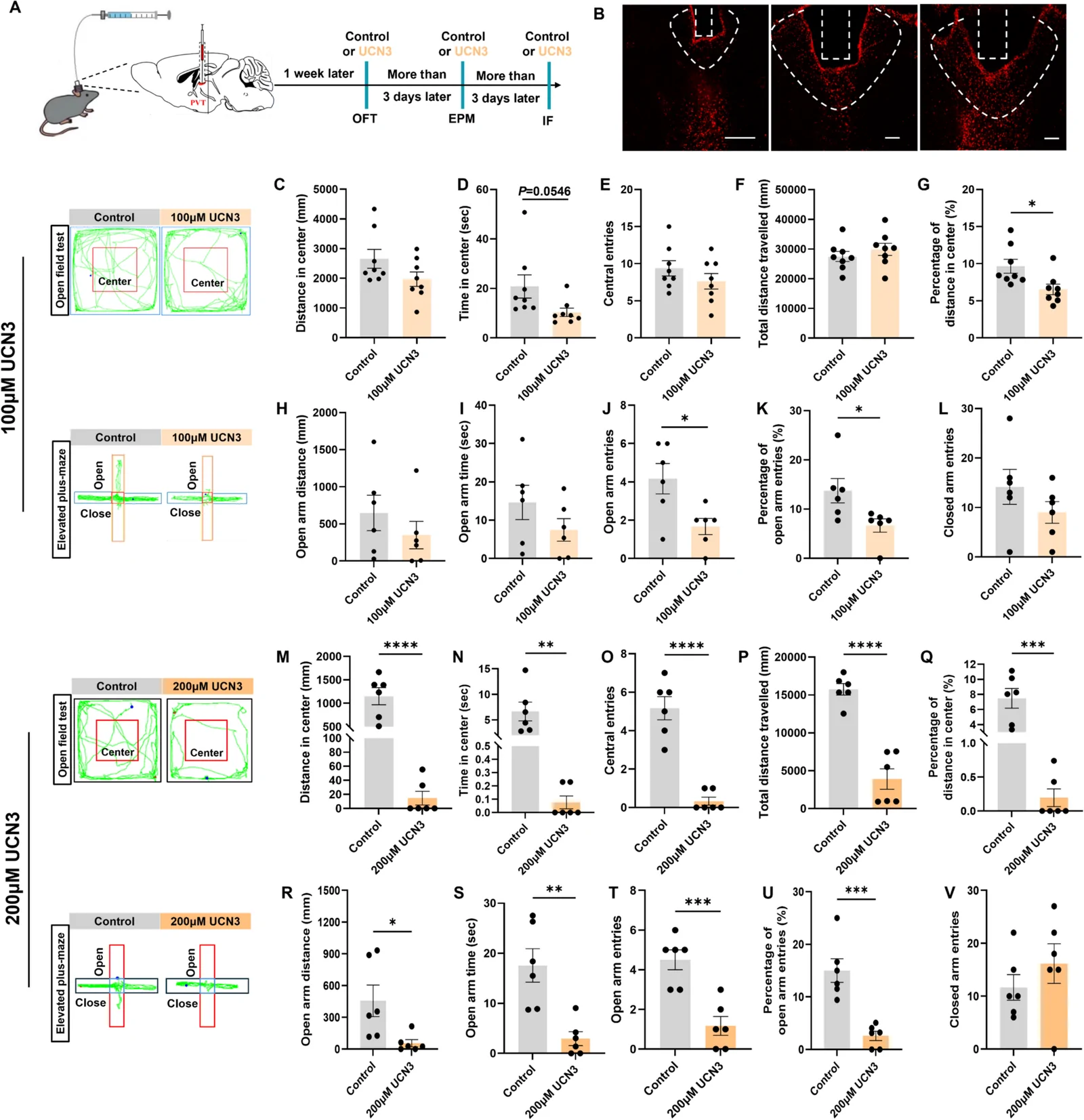
Microinfusion of CRFR2 agonists into the PVT produces anxiogenic effects. **A** Catheterization of the PVT brain ventricle and validation chart and PVT ventricular catheter injection of the selective CRFR2 agonist urocortin III (UCN3; 100 µM or 200 µM) or control (saline) at a rate of 0.2 l/min (0.5 µl total) directly before each behavior test. **B** Representative images of c-Fos-positive neurons. Scale bars, 100 μm and 200 μm. **C**–**G** Open field test: distance traveled in the center, time spent in the center area, number of central entries, total distance traveled, and percentage of distance traveled in the center (%) (*n* = 8 per group). Representative trajectory plots depict the behavioral patterns of a single representative subject. **H**–**L** Elevated plus-maze test: open arm distance, time spent in the open arm, number of open arm entries, percentage of entries into the open arm, and number of entries into the closed arm (*n* = 6 per group). Representative trajectory plots represent the behavior of a single representative subject. **M**–**Q** Open field test: distance traveled in the center, time spent in the center area, number of central entries, total distance traveled, and percentage of distance traveled in the center (%) (*n* = 8 per group). Representative trajectory plots depict the behavioral patterns of a single representative subject. **R**–**V** Elevated plus-maze test: Open arm distance, time spent in the open arm, number of open arm entries, percentage of open arm entries, and closed arm entries (*n* = 6 per group). Representative trajectory plots represent the behavior of a single representative subject. Data are presented as the mean ± SEM, and *P* values were determined using unpaired *t* tests (C-V). *P < 0.05, ** *P* < 0.01, ***P < 0.001, ****P < 0.0001

To explore the function of CRFR2 within the PVT, we performed microinjections of UCN3 (an agonist of CRFR2) into the PVT via preimplanted cannulas (Fig. 2A), and successful cannula implantation into the PVT was verified by representative images (Fig. 2B). Compared with control mice, 100 µM CRFR2 agonist-infused mice tended to be anxious, as they tended to spend less time (Fig. 2C) and traveled a shorter percentage of their distance in the center area (Fig. 2G) in the OFT, and they displayed fewer open arm entries and fewer open arm entries during the EPM test (Fig. 2J and K); the other parameters, such as distance traveled in the central area, central entries, and total distance in the OFT, distance traveled, and time spent in the open arm and closed arm entries in the EPM test, did not significantly differ (Fig. 2C, E and I, and 2 L).

Therefore, we performed another batch of behavioral tests using a higher dose of UCN3. We found that 200 µM UCN3 induced clear anxiety-like behaviors, characterized mainly by reduced distance traveled (Fig. 2M), shorter residence time (Fig. 2N), and fewer entries (Fig. 2O) into the center of the OFT, as well as significantly decreased distance (Fig. 2R), time (Fig. 2S), and number (Fig. 2T) in the open arms of the EPM test. We also observed that high-dose UCN3 reduced the total distance traveled by mice in the OFT (Fig. 2P); moreover, the percentage of distance traveled in the OFT center (Fig. 2Q) and the percentage of entries (Fig. 2U) into the EPM open arm also decreased. These results indicate that CRFR2 in the PVT is closely associated with anxiety-like behaviors in mice.

### The inhibition of CRFR2 in the PVT relieves heat-triggered anxiety-like behavior in mice, mainly in the OFT

**Fig. 3:**
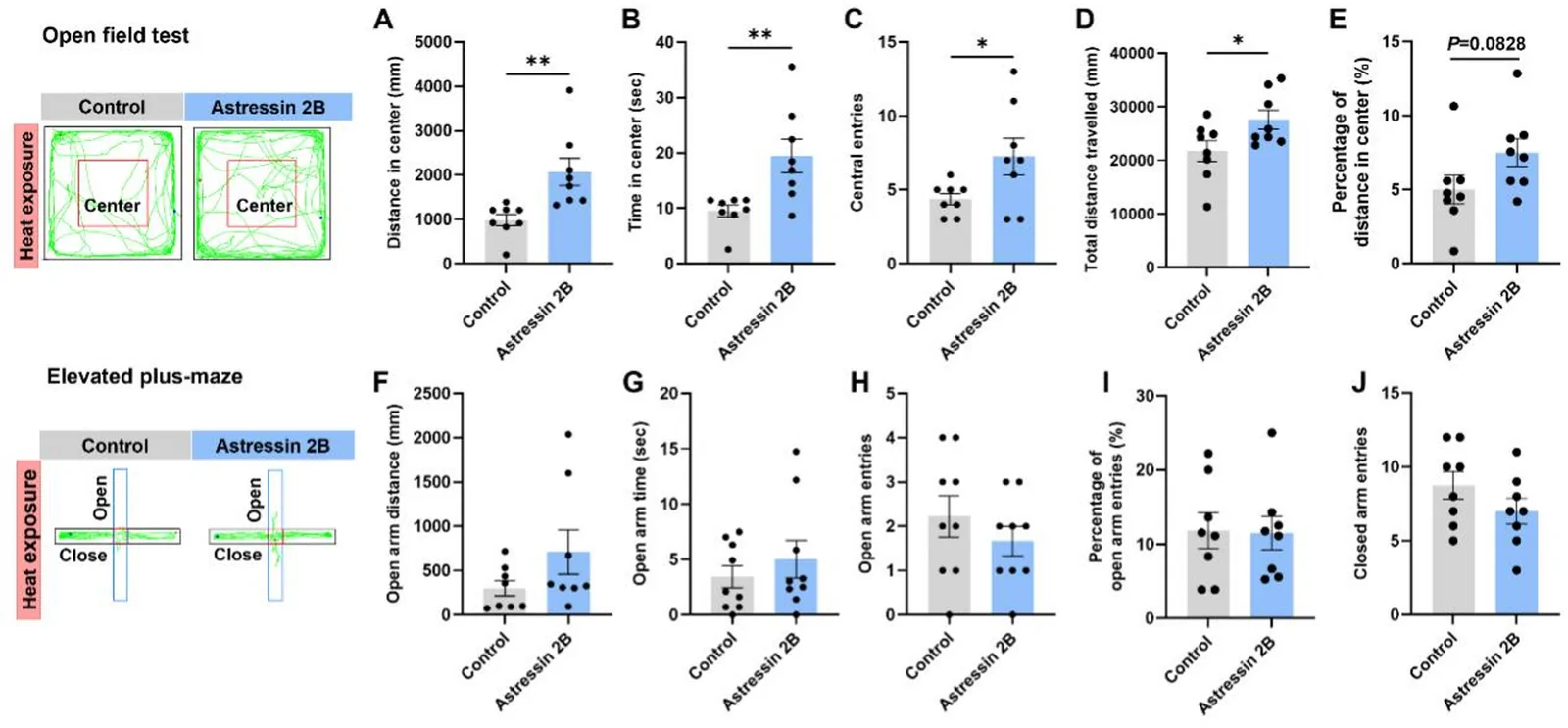
PVT CRFR2 inhibition alleviated heat-triggered anxiety-like behavior in mice primarily in the OFT. **A**–**E** Open field test: distance traveled in the center, time spent in the center area, number of central entries, total distance traveled, and percentage of distance traveled in the center (%) (*n* = 8 per group). Representative trajectory plots depict the behavioral patterns of a single representative subject. **F**‒**J** Elevated plus-maze test: distance traveled in the open arm, time spent in the open arm, number of open arm entries, percentage of open arm entries, and closed arm entries (*n* = 9 per group). Representative trajectory plots represent the behavior of a single representative subject. Data are presented as the mean ± SEM, and *P* values were determined using unpaired *t* tests (**A**–**J**). *P < 0.05, ** *P* < 0.01

Our results suggest that CRFR2 in the PVT may participate in the regulation of anxiety-like behavior induced by heat. To verify this hypothesis, the CRFR2 antagonist Astressin 2B was microinjected into the PVT of heat-exposed mice. Notably, CRFR2 inhibition significantly increased the distance traveled in the central area (Fig. 3A), time spent in the central area (Fig. 3B), and number of central entries (Fig. 3C) in the OFT of heat-exposed mice. Interestingly, inhibiting CRFR2 within the PVT tended to increase the percentage of distance traveled in the center of heat-exposed mice in the OFT (*P* = 0.082) (Fig. 3E), whereas the total distance traveled in the OFT also increased (Fig. 3D). In the EPM data, we failed to find any significant changes with Astressin 2B treatment (Fig. 3F and J). These results indicate that CRFR2 in the PVT specifically mediates anxiety-like behavior in mice in the OFT after heat exposure.

Importantly, the effect of CRFR2 inhibition was not limited to heat conditions: at room temperature, CRFR2 blockade in the PVT still promoted central exploration in the OFT (Fig. S3A–S3E) but did not alter EPM activity in mice (Fig. S3F–S3J). This consistency across thermal conditions suggests that CRFR2 in the PVT contributes to reducing the degree of fear of the unshielded central area of mice in the OFT.

### Mice with CRFR2 knockdown in the PVT exhibit reduced anxiety predominantly observed in the OFT after heat exposure

**Fig. 4:**
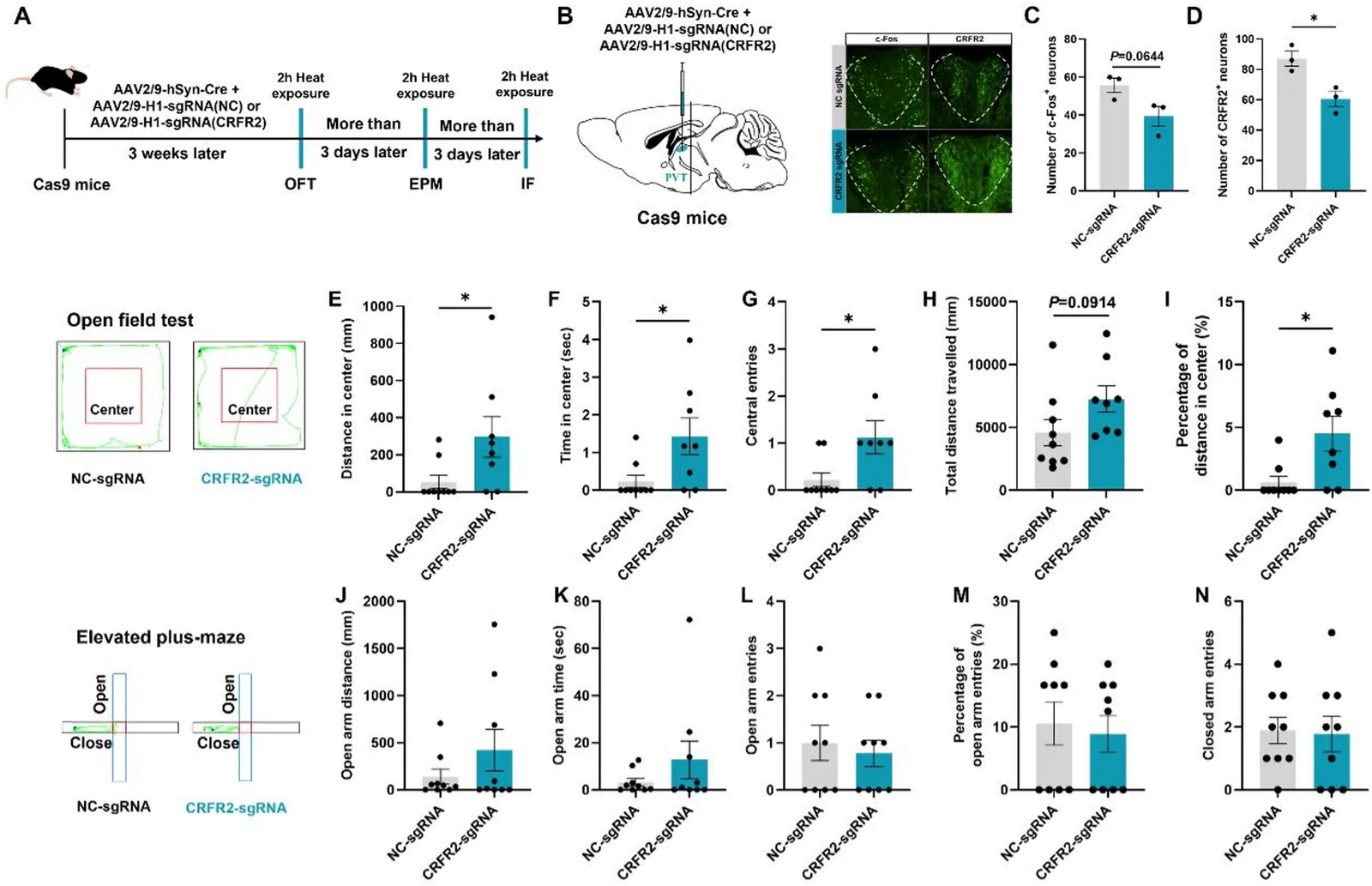
Knockdown of CRFR2 in the PVT decreases anxiety-like behavior mainly in the OFT in heat-exposed mice. **A** Paradigm of the mouse behavior assay. **B** Expression of the sgRNA (NC) or sgRNA (CRFR2) virus in the PVT neurons of Cas9 mice. **C**, **D** Number of PVT c-Fos- and CRFR2-positive neurons (green) in Cas9 mice (*n* = 3 per group). Scale bars, 200 μm. **E**–**I** Open field test: distance traveled in the center, time spent in the center area, number of central entries, total distance traveled, and percentage of distance traveled in the center (%) (*n* = 8–9 per group). Representative trajectory plots depict the behavioral patterns of a single representative subject. **J**‒**N** Elevated plus-maze test: distance traveled in the open arm, time spent in the open arm, number of open arm entries, percentage of open arm entries, and closed arm entries (*n* = 9 per group). Representative trajectory plots represent the behavior of a single representative subject. Data are presented as the mean ± SEM, and *P* values were determined using unpaired *t* tests (**C**‒**N**). *P < 0.05

To further validate the functional role of CRFR2 in the PVT (Fig. 4A), we used a genetic knockdown strategy: AAV2/9-hSyn-Cre and AAV2/9-CRFR2-sgRNA viruses were coinjected into the PVT of Cas9 mice to specifically achieve CRFR2 knockdown (Fig. 4B). As a result, the number of CRFR2-positive cells in the PVT was significantly reduced in the CRFR2 knockdown mice (Fig.4D). Additionally, the number of c-Fos-positive cells tended to decrease (Fig. 4C). Notably, this reduction in c-Fos and CRFR2 expression was also observed in knockdown mice under basal room temperature conditions (Fig. S4A–S4C).

Specifically, the distance traveled in the center (Fig. 4E), time spent in the center (Fig. 4F), number of central entries (Fig. 4G), and percentage of distance traveled in the center (Fig. 4I) of the mice during the OFT were all elevated, but the EPM test activity was not altered in heat-exposed mice (Fig. 4J and N). Furthermore, these mice exhibited less anxiety-like behavior even under normal room temperature conditions (Fig. S4).

### Knockdown of CRFR2 in PVT Vglut2 neurons alleviates OFT-dominant anxiety-like behavior in heat-exposed mice

**Fig. 5:**
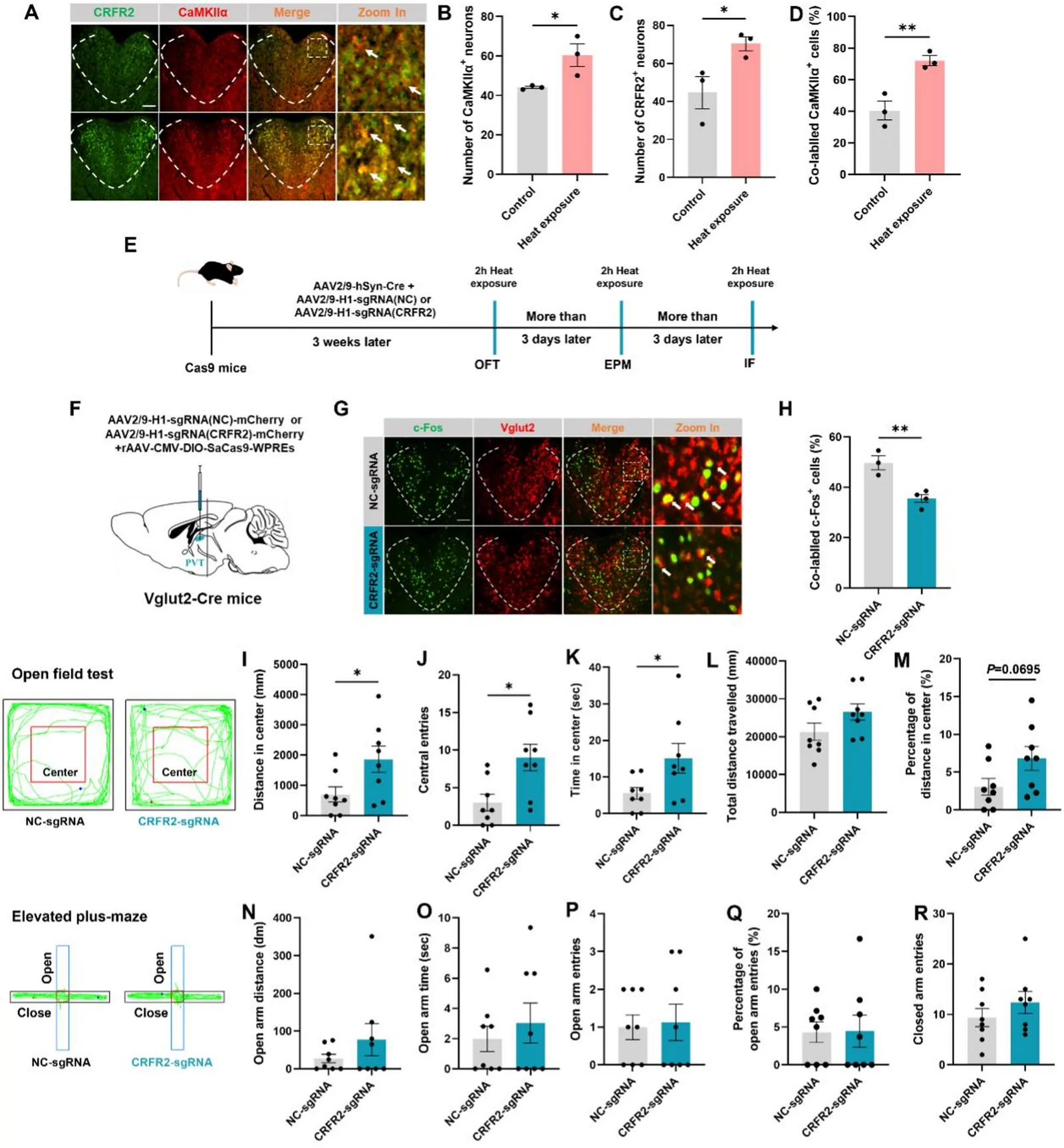
Knockdown of CRFR2 in Vglut2 neurons in the PVT mitigates OFT-dominant anxiety in heat-exposed mice. **A** Immunofluorescence images showing CaMKIIα neurons expressing CRFR2. Immunoreactivity of CRFR2 (green) and CaMKIIα (red) and merged images (yellow) after control or heat exposure in mice. White arrows indicate CaMKIIα-expressing neurons expressing CRFR2 (zoom in). Scale bars, 200 μm. **B**–**D** Number of PVT CaMKIIα-positive neurons (red) and CRFR2-positive neurons (green) and percentage of CaMKIIα neurons -expressing CRFR2 (yellow) in control or heat-exposed mice (*n* = 3 per group). **E** Mouse behavior paradigm. **F** Expression of sgRNA (NC) or sgRNA (CRFR2) and Cas9 in the PVT neurons of Vglut2 mice. **G** Immunofluorescence images showing Vglut2-expressing neurons expressing c-Fos. Immunoreactivity of c-Fos (green) and Vglut2 (red) and merged images (yellow) after heat exposure in mice. White arrows indicate Vglut2 neurons expressing c-Fos (zoom in). Scale bars, 200 μm. **H** Percentage of Vglut2 neurons expressing c-Fos (yellow) in the sgRNA (NC)- or sgRNA (CRFR2)-virus-injected Vglut2 mice (*n* = 3–4 per group). **I**–**M** Open field test: distance traveled in the center, time spent in the center area, number of central entries, total distance traveled, and percentage of distance traveled in the center (%) (*n* = 8 per group). Representative trajectory plots depict the behavioral patterns of a single representative subject. **N**–**R** Elevated plus-maze test: open arm distance, time spent in the open arm, number of open arm entries, percentage of open arm entries, and closed arm entries (*n* = 8 per group). Representative trajectory plots represent the behavior of a single representative subject. Data are presented as the mean ± SEM, and *P* values were determined using unpaired *t* tests (**B**–**D**, **H**–**R**). *P < 0.05, ** *P* < 0.01

Previous studies have demonstrated that the majority of neurons in the PVT are Vglut2 excitatory neurons, with CaMKIIα serving as a forebrain-specific promoter for glutamatergic neurons [34, 35]. In heat-exposed mice (Fig. 5A), the number of CaMKIIα-expressing neurons (Fig. 5B) and CRFR2-expressing neurons (Fig. 5C) and the proportion of CaMKIIα-expressing neurons expressing CRFR2 (Fig. 5D) significantly increased. To explore the specific neuronal populations within the PVT upon which CRFR2 acts, AAV-CRFR2-sgRNA and AAV-DIO-Cas9 viruses were used in Vglut2-Cre mice to knockdown CRFR2 specifically in PVT glutamate neurons (Fig. 5E–F). This strategy led to a reduction in c-Fos expression in Vglut2 neurons in the PVT of mice (Fig. 5G and H) and significantly diminished anxiety-like behavior in the OFT (Fig. 5I, J, K and M); similarly, this strategy did not affect the performance of the mice in the EPM test (Fig. 5N and R). Taken together, these results suggest that CRFR2 in the PVT may modulate anxiety-like behavior predominantly manifested in the OFT in heat-exposed mice by influencing the activity of Vglut2 neurons.

### Activation of PVT^Vglut2^ neurons induces anxiety primarily reflected in the OFT in mice

**Fig. 6:**
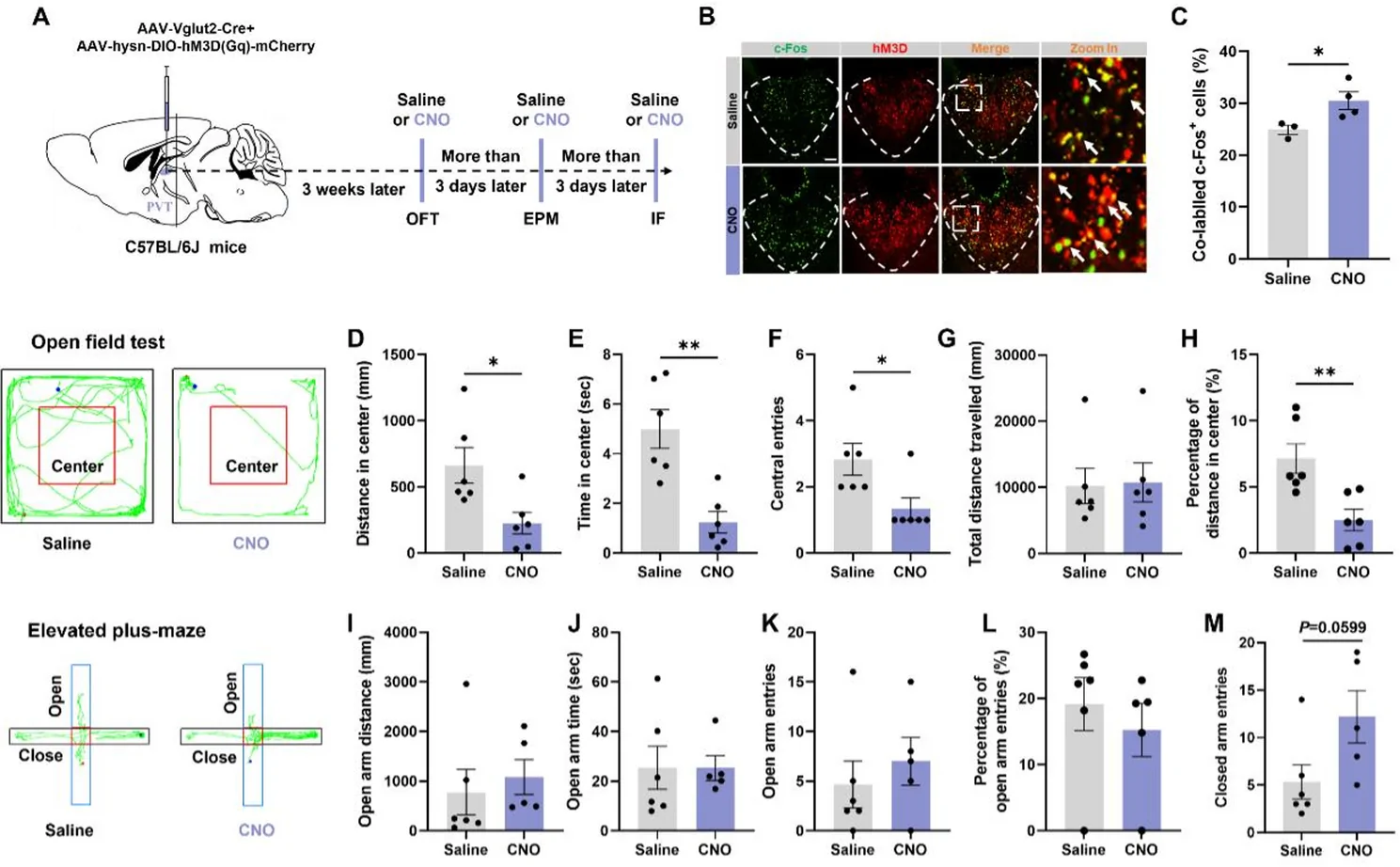
Activation of PVT^Vglut2^ neurons increases anxiety-like behavior primarily detected in the OFT in mice. **A** Expression of the hM3D (Gq) virus in PVT^Vglut2^ neurons and the paradigm of the mouse behavior assay. **B** Immunofluorescence images showing Vglut2-expressing neurons (labeled with hM3D) expressing c-Fos. Immunoreactivity of c-Fos (green), hM3D (red), and merged images (yellow). White arrows indicate Vglut2 neurons expressing c-Fos (zoom in). Scale bars, 200 μm. **C** Percentage of Vglut2 neurons expressing c-Fos (yellow) in saline- or CNO-injected WT mice (*n* = 3–4 per group). **D**–**H** Open field test: distance traveled in the center, time spent in the center area, number of central entries, total distance traveled, and percentage of distance traveled in the center (%) (*n* = 6 per group). Representative trajectory plots depict the behavioral patterns of a single representative subject. **I**–**M** Elevated plus-maze test: open arm distance, time spent in the open arm, number of open arm entries, percentage of open arm entries, and number of closed arm entries (*n* = 5–6 per group). Representative trajectory plots represent the behavior of a single representative subject. Data are presented as the mean ± SEM, and *P* values were determined using unpaired *t* tests (C‒M). *P < 0.05, ** *P* < 0.01

To investigate how PVT^Vglut2^ neurons influence anxiety-like behavior in mice, we injected a Cre-dependent hM3D (Gq) virus (AAV-DIO-hM3D-mCherry) into the PVT of Vglut2-Cre mice (Fig. 6A). An intraperitoneal injection of clozapine-N-oxide (CNO) successfully activated PVT^Vglut2^ neurons, as confirmed by the increased percentage of c-Fos-expressing neurons that coexpressed Vglut2 (Fig. 6B and C). This neuronal activation induced anxiety phenotypes characterized by decreased central area distance traveled (Fig. 6D), residence time (Fig. 6E), number of entries (Fig. 6F), and percentage of distance traveled in the center area (Fig. 6H) of the OFT. In the EPM test, the number of closed-arm entries tended to increase in PVT^Vglut2^-activated mice (Fig. 6M). The total distance traveled in the OFT (Fig. 6G), the distance traveled in the open arm, and the time spent in the open arm (Fig. 6I and L) did not differ. These behavioral changes mirror those observed following CRFR2 activation, suggesting that Vglut2 neurons and CRFR2 play convergent roles in PVT-mediated anxiety regulation, which is observed mainly in the OFT.

### Downregulation of PVT^Vglut2^ neuronal activity reverses heat-induced anxiety-like behavior manifested primarily in the OFT in mice

**Fig. 7:**
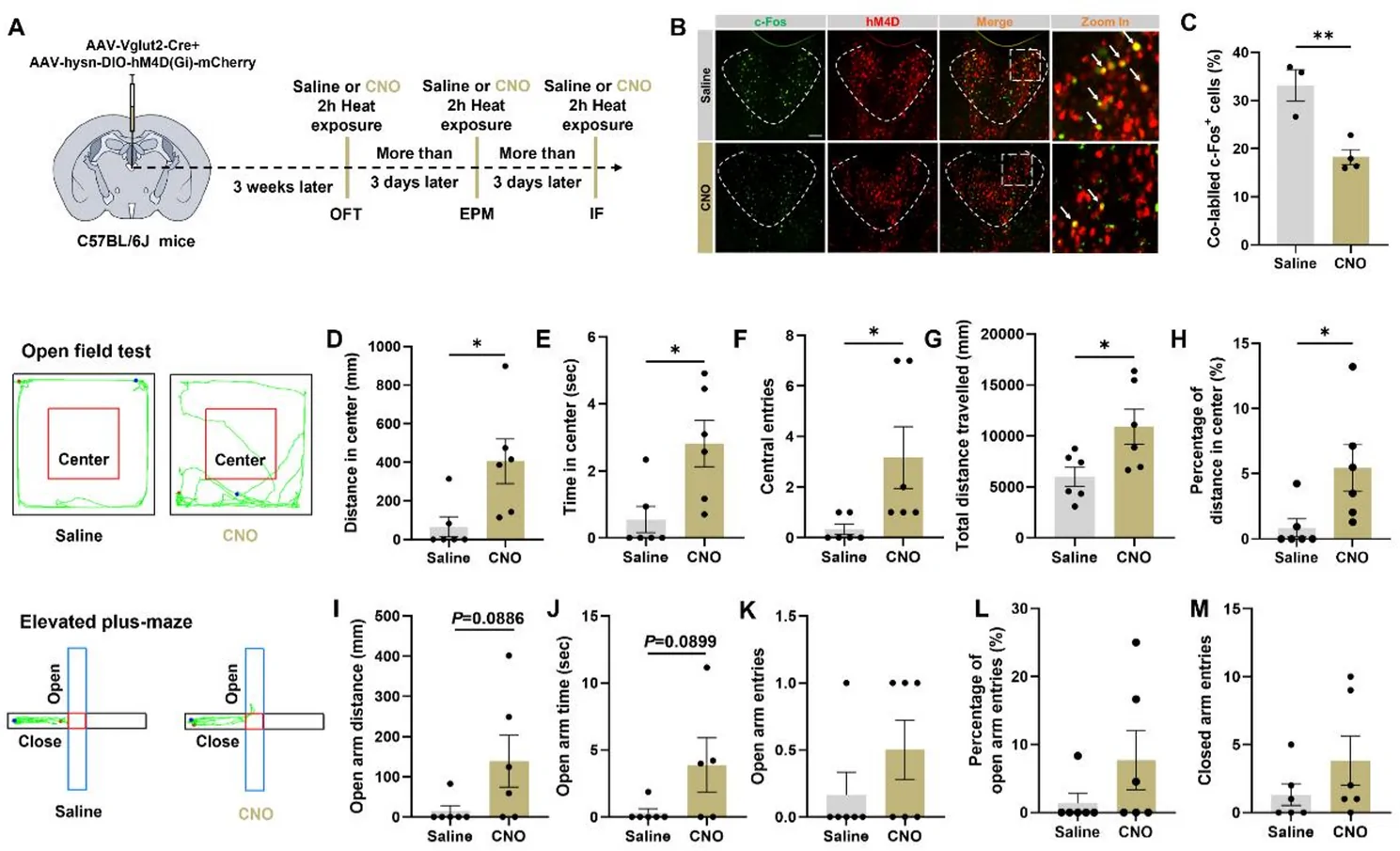
The inhibition of Vglut2 excitatory neurons in the PVT reduced anxiety-like behavior prominent in the OFT in heat-induced mice. **A** Expression of the hM4D (Gi) virus in PVT^Vglut2^ neurons and the results of a mouse behavior assay. **B** Immunofluorescence images showing Vglut2-expressing neurons (labeled with hM4D) expressing c-Fos. Immunoreactivity of c-Fos (green), hM4D (red), and merged images (yellow). White arrows indicate Vglut2 neurons expressing c-Fos (zoom in). Scale bars, 200 μm. **C** Percentage of Vglut2 neurons expressing c-Fos (yellow) in saline- or CNO-injected WT mice (*n* = 3 per group). **D**–**H** Open field test: distance traveled in the center, time spent in the center area, number of central entries, total distance traveled, and percentage of distance traveled in the center (%) (*n* = 6 per group). Representative trajectory plots depict the behavioral patterns of a single representative subject. **I**–**M** Elevated plus-maze test: open arm distance, time spent in the open arm, number of open arm entries, percentage of open arm entries, and closed arm entries (*n* = 6 per group). Representative trajectory plots represent the behavior of a single representative subject. Data are presented as the mean ± SEM, and *P* values were determined using unpaired *t* tests (C‒M). *P < 0.05, ** *P* < 0.01

To investigate whether inhibiting Vglut2 neurons in the PVT could reduce anxiety-like behavior in mice after heat exposure, we injected AAV-DIO-hM4D-mCherry (a Cre-dependent hM4D (Gi) virus) into the PVT of Vglut2-Cre mice (Fig. 7A), and the number of Vglut2 neurons expressing hM4D was inhibited by CNO administration under heat conditions (Fig. 7B and C). Anxiety-like behavior was evaluated at room temperature after saline or CNO injection. The mice showed no significant differences in anxiety-like behavior (Fig. S5). Subsequently, we evaluated anxiety-like behavior in AAV-hM4D-injected mice after heat exposure. These mice exhibited reduced anxiety-like behavior in the OFT, as evidenced by an increased travel distance (Fig. 7D), a greater number of center entries (Fig. 7E), a longer time spent in the center (Fig. 7F), an increased total travel distance (Fig. 7G), and a greater percentage of distance in the center (Fig. 7H). Additionally, in the EPM test, both the time spent in the open arm (Fig. 7I) and the distance traveled in the open arm (Fig. 7J) tended to increase without influencing the number of entries into the open arm or closed arm (Fig. 7K and M).

## Discussion

Previous research has demonstrated that animals facing acute heat stress exhibit an initial emotional response that precedes thermoregulatory mechanisms [36]. Exposure to hot or other adverse environmental conditions disrupts the HPA axis [37], leading to prolonged elevation of glucocorticoids and increased vulnerability to stress-related pathologies such as anxiety and depression [38, 39]. In our study, heat exposure was associated with reduced activity in the central area of the open field and the open arms of the elevated plus maze, as reflected by residence time, enter frequency, and movement distance. These behavioral alterations are indicative of heightened anxiety-like phenotypes in mice, which align with prior observations [3, 6]. Critically, we identified a pronounced sex difference: heat-induced anxiety-like behavior was exclusively observed in male mice, suggesting greater sensitivity in male mice.

As a pivotal regulator of the stress response, the CRF system plays a central role in modulating animal behavior. Evidence highlights CRFR2 as a mediator of sex-specific stress responses [24]. Our findings revealed that CRFR2 expression in the classical PVH remained unaltered following heat exposure. In contrast, CRFR2 expression in the PVT tended to increase in heat-exposed male mice. As a key component of the thalamic midline nuclear complex, the unique anatomical positioning and neural circuitry of the PVT endows it with the capacity to integrate diverse neural and endocrine signals to modulate behavioral outputs [40, 41].

On the other hand, the CRFR2 ligand urocortin (UCN), a hypothalamic CRF-related peptide with high-affinity binding to CRFR2, regulates downstream signaling via the PKB/AKT and CREB pathways, which are critical for synaptic plasticity [42, 43]. Notably, heat exposure suppressed the phosphorylation of AKT and CREB in the PVT of male mice, which is similar to our previous findings that elevated CRFR2 levels are associated with the suppression of the AKT/CREB pathway under cold stress [27].

To characterize the specific role of CRFR2 in PVT under heat conditions, PVT cannulation (local drug delivery) and CRFR2 knockdown models were used for subsequent experiments. Intra-PVT injection of a CRFR2 agonist in mice induced anxiety-like behavior in the OFT, whereas intra-PVT injection of a CRFR2 antagonist in heat-exposed mice mitigated these effects predominantly observed in the OFT, directly linking CRFR2 activity to heat-induced anxiety. Knockdown of CRFR2 in the PVT also attenuated OFT-dominant anxiety phenotypes in heat-exposed mice, corroborating its role as an anxiety signaling pathway. Importantly, although heat exposure and CRFR2 manipulation also affected locomotor activity in the OFT, the percentage of entries into the central zone still exhibited clear anxiety-related changes. These results indicate that inhibiting CRFR2 in the PVT not only mediates heat-induced anxiety-like behaviors primarily in the OFT but also modulates basal locomotor activity in mice. Specifically, CRFR2 in the PVT facilitates locomotor activity, thereby promoting hyperactivity in anxiety-like mouse models. To better distinguish thermoregulation-related behavioral suppression from authentic anxiety-associated affective changes, future studies may employ alternative behavioral assays that are minimally reliant on locomotion, such as defensive burying, foot shock-evoked escape tests and predator avoidance paradigms. These approaches offer important insights for further research on PVT-regulated emotional responses to heat. Collectively, these findings demonstrate that CRFR2 in the PVT is indeed involved in regulating anxiety-like behaviors predominantly observed in the OFT in mice exposed to heat.

On the other hand, the most influenced behavior in our study seems to be the exploration of mice in the central zone in the OFT; however, several models fail to show alternation in behavior in the EPM test. Anxiety-like behavior in the EPM does not seem to be the main point affected by CRFR2 in the PVT. The EPM is based on the decision-making conflict between “exploring open high-risk areas” and “taking shelter in closed safe areas”, and the challenge is stronger in the EPM than in the open field. Notably, closed-arm entries have also been validated as a reliable index of general locomotor activity [44]. We report similar findings in our previous publication in which the effects of TLR4 on Tph2 neurons in the DRN were studied [45], and anxiety-like behavior in the OFT but not in the EPM was the target. Additionally, compared with that in the OFT, Fos expression in the thalamus has been reported to respond less strongly to the EPM test [46]. Therefore, we suggest that merely inhibiting CRFR2 in the PVT is not sufficient to completely reverse the anxiety-like behaviors caused by heat exposure in the elevated plus-maze test.

Receptors serve as critical mediators of neuronal function, integrating signals from neurotransmitters, hormones, and environmental stimuli to modulate excitability, synaptic plasticity, and circuit dynamics. Focusing on the PVT—a key node in the dorsal midline thalamus—its neuronal composition is dominated by Vglut2 excitatory neurons, with sparse GABAergic neurons [34, 47]. These glutamatergic neurons, which release glutamate as their primary neurotransmitter, form extensive projections across the central nervous system and are critical for emotional behavior [48–51]. Preclinical evidence links Vglut2 neurons in the PVT to diverse functions: regulating emotional behavior via projections to the medial insular cortex [52], modulating stress-induced reward seeking through the PVT project to the BNST^CRF^ circuit [53], and mediating pain-anxiety comorbidity in Parkinsonian models [54]. We hypothesized that the key to anxiety induced by heat exposure is the expression of CRFR2 in Vglut2 neurons, given that PVT neurons exhibit increased activity and that the expression of CRFR2 tends to increase. With CaMKIIα—a specific marker of forebrain glutamatergic neurons and a key regulator of Ca²⁺-dependent synaptic plasticity [35]—we detected high colocalization of CRFR2 with CaMKIIα in PVT neurons, confirming that CRFR2 is primarily expressed in excitatory neurons in this region. Functional validation revealed that genetic knockdown of CRFR2 in PVT^Vglut2^ neurons attenuated heat-induced anxiety-like behavior in the OFT. Furthermore, the use of chemical genetics techniques to activate Vglut2 neurons in the PVT increased anxiety-like behavior, which was reflected mainly in the OFT in mice—matching behaviors observed in both heat-exposed mice and when CRFR2 was activated in the PVT. Conversely, the inhibition of Vglut2 neuronal activity or CRFR2 expression in the PVT abolished heat-induced anxiety primarily observed in the OFT, establishing a causal relationship between CRFR2 signaling in PVT glutamatergic neurons and behavioral vulnerability.

## Conclusions

In summary, our results demonstrate that heat exposure induced anxiety-like behavior specifically in male mice. CRFR2 levels and activity in Vglut2 neurons within the PVT contribute to the development of anxiety-like behavior in the OFT following heat exposure in male mice. Knockdown of CRFR2 or inhibition of Vglut2 neurons significantly attenuated heat-induced anxiety-like responses in the open field test.

## Supplementary Information

Below is the link to the electronic supplementary material.

Supplementary Material 1

## Data Availability

No datasets were generated or analysed during the current study.

## Abbreviations

AAV: Adeno-associated virus
ACTH: Adrenocorticotropic hormone
AKT/PKB: Protein kinase B
BNST: Bed nucleus of the stria terminalis
CeA: Central amygdala
CREB: CAMP-response element binding protein
CRF: Corticotropin releasing factor
CRFR2: Corticotropin releasing hormone receptor 2
CRH: Corticotropin releasing hormone
DRN: Dorsal raphe nucleus
EPM: Elevated plus maze
GC: Glucocorticoid
HPA: Hypothalamic‒pituitary‒adrenal axis
NAc: nucleus accumbens
OFT: Open field test
PAG: Periaqueductal gray
PVH: Paraventricular hypothalamic nucleus
PVT: Paraventricular nucleus of the thalamus
SCN: Suprachiasmatic nucleus
UCN: Urocortin
UCN3: Urocortin III

## References

[1] Chen SF, Chuang CY, Chao CC, Yang YH, Chu CY, Yao CY, Su YC, Huang YH, Liao RM. Task-Dependent Differences in Operant Behaviors of Rats With Acute Exposure to High Ambient Temperature: A Potential Role of Hippocampal Dopamine Reuptake Transporters. Front Behav Neurosci. 2019;13:15. 10.3389/fnbeh.2019.00015. PMC6369190 30778291

[2] Gruene TM, Lipps J, Rey CD, Bouck A, Shansky RM. Heat exposure in female rats elicits abnormal fear expression and cellular changes in prefrontal cortex and hippocampus. Neurobiol Learn Mem. 2014;115:38–42. 10.1016/j.nlm.2014.05.004. 24859751

[3] Mete F, Kilic E, Somay A, Yilmaz B. Effects of heat stress on endocrine functions & behaviour in the pre-pubertal rat. Indian J Med Res. 2012;135(2):233–9. PMC3336856 22446867

[4] Lieberman HR, Georgelis JH, Maher TJ, Yeghiayan SK. Tyrosine prevents effects of hyperthermia on behavior and increases norepinephrine. Physiol Behav. 2005;84(1):33–8. 10.1016/j.physbeh.2004.10.023. 15642604

[5] Yeghiayan SK, Georgelis JH, Maher TJ, Lieberman HR. Beneficial effects of a protein free, high carbohydrate meal on rat coping behavior and neurotransmitter levels during heat stress. Nutr Neurosci. 2004;7(5–6):335–40. 10.1080/10284150400017330. 15682930

[6] Erfani M, Ghazi Tabatabaei Z, Sadigh-Eteghad S, Farokhi-Sisakht F, Farajdokht F, Mahmoudi J, Karimi P, Nasrolahi A. Rosa canina L. methanolic extract prevents heat stress-induced memory dysfunction in rats. Exp Physiol. 2019;104(10):1544–54. 10.1113/ep087535. 31297904

[7] Sharma HS, Sharma A, Mössler H, Muresanu DF. Neuroprotective effects of cerebrolysin, a combination of different active fragments of neurotrophic factors and peptides on the whole body hyperthermia-induced neurotoxicity: modulatory roles of co-morbidity factors and nanoparticle intoxication. Int Rev Neurobiol. 2012;102:249–76. 10.1016/b978-0-12-386986-9.00010-7. 22748833

[8] Dong HW, Petrovich GD, Watts AG, Swanson LW. Basic organization of projections from the oval and fusiform nuclei of the bed nuclei of the stria terminalis in adult rat brain. J Comp Neurol. 2001;436(4):430–55. 10.1002/cne.1079. 11447588

[9] Igelstrom KM, Herbison AE, Hyland BI. Enhanced c-Fos expression in superior colliculus, paraventricular thalamus and septum during learning of cue-reward association. Neuroscience. 2010;168(3):706–14. 10.1016/j.neuroscience.2010.04.018. 20399252

[10] Kirouac GJ. Placing the paraventricular nucleus of the thalamus within the brain circuits that control behavior. Neurosci Biobehav Rev. 2015;56:315–29. 10.1016/j.neubiorev.2015.08.005. 26255593

[11] Campus P, Covelo IR, Kim Y, Parsegian A, Kuhn BN, Lopez SA, Neumaier JF, Ferguson SM, Solberg Woods LC, Sarter M, Flagel SB. The paraventricular thalamus is a critical mediator of top-down control of cue-motivated behavior in rats. Elife. 2019. 10.7554/eLife.49041. PMC6739869 31502538

[12] Kirouac GJ, Parsons MP, Li S. Orexin (hypocretin) innervation of the paraventricular nucleus of the thalamus. Brain Res. 2005;1059(2):179–88. 10.1016/j.brainres.2005.08.035. 16168969

[13] Zhang J, Chen D, Sweeney P, Yang Y. An excitatory ventromedial hypothalamus to paraventricular thalamus circuit that suppresses food intake. Nat Commun. 2020;11(1):6326. 10.1038/s41467-020-20093-4. PMC7728757 33303759

[14] Otis JM, Zhu M, Namboodiri VMK, Cook CA, Kosyk O, Matan AM, Ying R, Hashikawa Y, Hashikawa K, Trujillo-Pisanty I, Guo J, Ung RL, Rodriguez-Romaguera J, Anton ES, Stuber GD. Paraventricular Thalamus Projection Neurons Integrate Cortical and Hypothalamic Signals for Cue-Reward Processing. Neuron. 2019;103(3):423–31. 10.1016/j.neuron.2019.05.018. PMC6773659 31196673

[15] Zhou K, Xu H, Lu S, Jiang S, Hou G, Deng X, He M, Zhu Y. Reward and aversion processing by input-defined parallel nucleus accumbens circuits in mice. Nat Commun. 2022;13(1):6244. 10.1038/s41467-022-33843-3. PMC9587247 36271048

[16] Deng J, Chen L, Liu CC, Liu M, Guo GQ, Wei JY, Zhang JB, Fan HT, Zheng ZK, Yan P, Zhang XZ, Zhou F, Huang SX, Zhang JF, Xu T, Xie JD, Xin WJ. Distinct Thalamo-Subcortical Circuits Underlie Painful Behavior and Depression-Like Behavior Following Nerve Injury. Adv Sci (Weinh). 2024;11(34):e2401855. 10.1002/advs.202401855. PMC11425852 38973158

[17] Moga MM, Moore RY. Organization of neural inputs to the suprachiasmatic nucleus in the rat. J Comp Neurol. 1997;389(3):508–34. https://onlinelibrary.wiley.com/doi/10.1002/(SICI)1096-9861(19971222)389:3%3C508::AID-CNE11%3E3.0.CO;2-H. 10.1002/(sici)1096-9861(19971222)389:3<508::aid-cne11>3.0.co;2-h 9414010

[18] Penzo MA, Robert V, Tucciarone J, De Bundel D, Wang M, Van Aelst L, Darvas M, Parada LF, Palmiter RD, He M, Huang ZJ, Li B. The paraventricular thalamus controls a central amygdala fear circuit. Nature. 2015;519(7544):455–9. 10.1038/nature13978. PMC4376633 25600269

[19] Dieterich KD, Lehnert H, De Souza EB. Corticotropin-releasing factor receptors: an overview. Exp Clin Endocrinol Diabetes. 1997;105(2):65–82. 10.1055/s-0029-1211730. 9137936

[20] Zhang N, Zhao S, Ma Y, Xiao Z, Xue B, Dong Y, Wang Q, Xu H, Zhang X, Wang Y. Hyperexcitation of ovBNST CRF neurons during stress contributes to female-biased expression of anxiety-like avoidance behaviors. Sci Adv. 2024;10(19):eadk. 10.1126/sciadv.adk7636. PMC11086623 38728397

[21] Zhu X, Zhang C, Hu Y, Wang Y, Xiao S, Zhu Y, Sun H, Sun J, Xu C, Xu Y, Chen Y, He X, Liu B, Liu J, Du J, Liang Y, Liu B, Li X, Jiang Y, Shen Z, Shao X, Fang J. Modulation of Comorbid Chronic Neuropathic Pain and Anxiety-Like Behaviors by Glutamatergic Neurons in the Ventrolateral Periaqueductal Gray and the Analgesic and Anxiolytic Effects of Electroacupuncture. eNeuro. 2024;11(8). 10.1523/eneuro.0454-23.2024. PMC11360982 39084906

[22] Henckens MJ, Deussing JM, Chen A. Region-specific roles of the corticotropin-releasing factor-urocortin system in stress. Nat Rev Neurosci. 2016;17(10):636–51. 10.1038/nrn.2016.94. 27586075

[23] Bale TL, Contarino A, Smith GW, Chan R, Gold LH, Sawchenko PE, Koob GF, Vale WW, Lee KF. Mice deficient for corticotropin-releasing hormone receptor-2 display anxiety-like behaviour and are hypersensitive to stress. Nat Genet. 2000;24(4):410–4. 10.1038/74263. 10742108

[24] Piccin A, Contarino A. Sex-linked roles of the CRF(1) and the CRF(2) receptor in social behavior. J Neurosci Res. 2020;98(8):1561–74. 10.1002/jnr.24629. 32468598

[25] Kishimoto T, Radulovic J, Radulovic M, Lin CR, Schrick C, Hooshmand F, Hermanson O, Rosenfeld MG, Spiess J. Deletion of crhr2 reveals an anxiolytic role for corticotropin-releasing hormone receptor-2. Nat Genet. 2000;24(4):415–9. 10.1038/74271. 10742109

[26] Donner NC, Lowry CA. Sex differences in anxiety and emotional behavior. Pflugers Arch. 2013;465(5):601–26. 10.1007/s00424-013-1271-7. PMC3805826 23588380

[27] Zhou Y, Zhu S, Ye S, Ye Z, Chen W, Li J, Shu G, Wang S, Zhu C, Wu R, Jiang Q, Wang L. Central corticotropin releasing hormone receptor 2 may participate in sex differential regulation of cold-evoked eating behavior. Neuroscience. 2025;566:169–76. 10.1016/j.neuroscience.2024.12.053. 39733820

[28] Y O, T I, T Y, I T-K, T Y, M Y. The serotonergic projection from the median raphe nucleus to the ventral hippocampus is involved in the retrieval of fear memory through the corticotropin-releasing factor type 2 receptor. Neuropsychopharmacology. 2010;35(6):1271–8. 10.1038/npp.2009.229. PMC3055345 20072117

[29] L T, B G-VM JS. Importance of CRF receptor-mediated mechanisms of the bed nucleus of the stria terminalis in the processing of anxiety and pain. Neuropsychopharmacology. 2014;39(11):2633–45. 10.1038/npp.2014.117. PMC4207343 24853772

[30] C LCHWYLXFSZKXXWZSGSSWPGXZ. Corticotropin-releasing factor receptor type 2 in the midbrain critically contributes to the hedonic feeding behavior of mice under heat stress. Biochem Biophys Res Commun. 2022;602:77–83. 10.1016/j.bbrc.2022.02.109. 35255437

[31] Décarie-Spain L, Sharma S, Hryhorczuk C, Issa-Garcia V, Barker PA, Arbour N, Alquier T, Fulton S. Nucleus accumbens inflammation mediates anxiodepressive behavior and compulsive sucrose seeking elicited by saturated dietary fat. Mol Metab. 2018;10:1–13. 10.1016/j.molmet.2018.01.018. PMC5985233 29454579

[32] Demers G, Roy J, Machuca-Parra AI, Dashtehei Pour Z, Bairamian D, Daneault C, Rosiers CD, Ferreira G, Alquier T, Fulton S. Fish oil supplementation alleviates metabolic and anxiodepressive effects of diet-induced obesity and associated changes in brain lipid composition in mice. Int J Obes (Lond). 2020;44(9):1936–45. 10.1038/s41366-020-0623-6. 32546855

[33] Sharma S, Fulton S. Diet-induced obesity promotes depressive-like behaviour that is associated with neural adaptations in brain reward circuitry. Int J Obes (Lond). 2013;37(3):382–9. 10.1038/ijo.2012.48. 22508336

[34] Iglesias AG, Flagel SB. The Paraventricular Thalamus as a Critical Node of Motivated Behavior via the Hypothalamic-Thalamic-Striatal Circuit. Front Integr Neurosci. 2021;15:706713. 10.3389/fnint.2021.706713. PMC8250420 34220458

[35] Zhu H, Wang N, Yao L, Chen Q, Zhang R, Qian J, Hou Y, Guo W, Fan S, Liu S, Zhao Q, Du F, Zuo X, Guo Y, Xu Y, Li J, Xue T, Zhong K, Song X, Huang G, Xiong W. Moderate UV Exposure Enhances Learning and Memory by Promoting a Novel Glutamate Biosynthetic Pathway in the Brain. Cell. 2018;173(7):1716–27. 10.1016/j.cell.2018.04.014. 29779945

[36] Collins KJ, Weiner JS. Endocrinological aspects of exposure to high environmental temperatures. Physiol Rev. 1968;48(4):785–839. 10.1152/physrev.1968.48.4.785. 4881390

[37] Borodovitsyna O, Flamini MD, Chandler DJ. Acute Stress Persistently Alters Locus Coeruleus Function and Anxiety-like Behavior in Adolescent Rats. Neuroscience. 2018;373:7–19. 10.1016/j.neuroscience.2018.01.020. 29341884

[38] Keller J, Gomez R, Williams G, Lembke A, Lazzeroni L, Murphy GM Jr., Schatzberg AF. HPA axis in major depression: cortisol, clinical symptomatology and genetic variation predict cognition. Mol Psychiatry. 2017;22(4):527–36. 10.1038/mp.2016.120. PMC5313380 27528460

[39] Yuan WX, Wu XJ, Yang FX, Shang XH, Zhang LL. Effects of ginseng root saponins on brain monoamines and serum corticosterone in heat-stressed mice. Zhongguo Yao Li Xue Bao. 1989;10(6):492–6. 2641845

[40] Matzeu A, Zamora-Martinez ER, Martin-Fardon R. The paraventricular nucleus of the thalamus is recruited by both natural rewards and drugs of abuse: recent evidence of a pivotal role for orexin/hypocretin signaling in this thalamic nucleus in drug-seeking behavior. Front Behav Neurosci. 2014;8:117. 10.3389/fnbeh.2014.00117. PMC3982054 24765071

[41] Millan EZ, Ong Z, McNally GP. Paraventricular thalamus: Gateway to feeding, appetitive motivation, and drug addiction. Prog Brain Res. 2017;235:113–37. 10.1016/bs.pbr.2017.07.006. 29054285

[42] Brar BK, Stephanou A, Knight R, Latchman DS. Activation of protein kinase B/Akt by urocortin is essential for its ability to protect cardiac cells against hypoxia/reoxygenation-induced cell death. J Mol Cell Cardiol. 2002;34(4):483–92. 10.1006/jmcc.2002.1529. 11991736

[43] Hauger RL, Risbrough V, Brauns O, Dautzenberg FM. Corticotropin releasing factor (CRF) receptor signaling in the central nervous system: new molecular targets. CNS Neurol Disord Drug Targets. 2006;5(4):453–79. 10.2174/187152706777950684. PMC1925123 16918397

[44] RJ R, NJ J. Factor analysis of spatiotemporal and ethological measures in the murine elevated plus-maze test of anxiety. Pharmacol Biochem Behav. 1995;52(2):297–303. 10.1016/0091-3057(95)00138-m. 8577794

[45] K X YLSZ, X XFLC, C WZSGSSW. TLR4 in Tph2 neurons modulates anxiety-related behaviors in a sex-dependent manner. Neuropharmacology. 2022. 10.1016/j.neuropharm.2022.109175. 35787402

[46] O NSPS. Neurobiological correlates of high (HAB) versus low anxiety-related behavior (LAB): differential Fos expression in HAB and LAB rats. Biol Psychiatry. 2004;55(7):715–23. 10.1016/j.biopsych.2003.10.021. 15039000

[47] Arcelli P, Frassoni C, Regondi MC, De Biasi S, Spreafico R. GABAergic neurons in mammalian thalamus: a marker of thalamic complexity? Brain Res Bull. 1997;42(1):27–37. 10.1016/s0361-9230(96)00107-4. 8978932

[48] Barbano MF, Wang HL, Zhang S, Miranda-Barrientos J, Estrin DJ, Figueroa-González A, Liu B, Barker DJ, Morales M. VTA Glutamatergic Neurons Mediate Innate Defensive Behaviors. Neuron. 2020;107(2):368–82. 10.1016/j.neuron.2020.04.024. PMC7381361 32442399

[49] Borkar CD, Stelly CE, Fu X, Dorofeikova M, Le QE, Vutukuri R, Vo C, Walker A, Basavanhalli S, Duong A, Bean E, Resendez A, Parker JG, Tasker JG, Fadok JP. Top-down control of flight by a non-canonical cortico-amygdala pathway. Nature. 2024;625(7996):743–9. 10.1038/s41586-023-06912-w. PMC10878556 38233522

[50] Luo YJ, Ge J, Chen ZK, Liu ZL, Lazarus M, Qu WM, Huang ZL, Li YD. Ventral pallidal glutamatergic neurons regulate wakefulness and emotion through separated projections. iScience. 2023;26(8):107385. 10.1016/j.isci.2023.107385. PMC10440712 37609631

[51] Zhang GW, Shen L, Tao C, Jung AH, Peng B, Li Z, Zhang LI, Tao HW. Medial preoptic area antagonistically mediates stress-induced anxiety and parental behavior. Nat Neurosci. 2021;24(4):516–28. 10.1038/s41593-020-00784-3. PMC8328037 33526942

[52] Campbell RGA, Walker EJ, SCn LCS, Muthmainah M, F SK AMG, O’Shea MJ, He S, Dayas CV, Andrews ZB, Lawrence AJ, Brown RM. A paraventricular thalamus to insular cortex glutamatergic projection gates emotional stress-induced binge eating in females. Neuropsychopharmacology. 2023;48(13):1931–40. 10.1038/s41386-023-01665-6. PMC10584903 37474763

[53] Levine OB, Skelly MJ, Miller JD, Rivera-Irizarry JK, Rowson SA, DiBerto JF, Rinker JA, Thiele TE, Kash TL, Pleil KE. The paraventricular thalamus provides a polysynaptic brake on limbic CRF neurons to sex-dependently blunt binge alcohol drinking and avoidance behavior in mice. Nat Commun. 2021;12(1):5080. 10.1038/s41467-021-25368-y. PMC8382748 34426574

[54] Zhang Y, Jiang Y, Yu Z, Li Y, Lin X, Weng Y, Guo Z, Hu H, Shao W, Yu G, Zheng F, Cai P, Li H, Wu S. VGluT2 neuron subtypes in the paraventricular thalamic nucleus regulate depression in paraquat-induced Parkinson’s disease. J Hazard Mater. 2024. 10.1016/j.jhazmat.2024.134559. 38735189

