# Supplementary material for: CRFR2 in paraventricular thalamus (PVT) mediates heat-induced anxiety in male mice
Source: Cell Biosci. 2026 Jul 25;16:115. doi: 10.1186/s13578-026-01624-w (PMC13688743; doi:10.1186/s13578-026-01624-w)
Supplement: Supplementary file 1 [file 13578_2026_1624_MOESM1_ESM.docx]

**Fig. S1. Effect of heat exposure on excitability of PVH neurons, expression and signaling pathway of CRFR2 in mice.**

(A) c-Fos and CRFR2 expression in the PVH (n = 3-4 per group, 4-6 brain slices per mouse). Scale bars, 200 μm.

(B) CRFR2 protein, phosphorylated AKT and CREB level in the PVH (n = 5-6 per group). Data presented as mean ± SEM, *P* value determined using multiple unpaired *t* tests (A-B). ** *P* < 0.01.

**Fig. S2. Representative immunofluorescence images, OFT and EPM trajectory plots of mice with inaccurate positioned injections.**

(A) Representative immunofluorescence images, OFT and EPM trajectory plots of mice with incorrectly positioned PVT ventricular catheters for UCN3 injection. Scale bars, 100 μm.

(B) Representative immunofluorescence images, OFT and EPM trajectory plots of heat-exposed mice with incorrectly positioned PVT ventricular catheters for Astressin 2B injection. Scale bars, 100 μm.

(C) Representative immunofluorescence images, OFT and EPM trajectory plots of heat-exposed Cas9 mice with incorrectly positioned PVT injections of CRFR2-sgRNA. Scale bars, 200 μm.

(D) Representative immunofluorescence images, OFT and EPM trajectory plots of heat-exposed Vglut2-Cre mice with incorrectly positioned PVT injections of CRFR2-sgRNA. Scale bars, 200 μm.

(E) Representative immunofluorescence images, OFT and EPM trajectory plots of mice with incorrectly positioned PVT injections of hM3D(Gq). Scale bars, 200 μm.

(F) Representative immunofluorescence images, OFT and EPM trajectory plots of heat-exposed mice with incorrectly positioned PVT injections of hM4D(Gi). Scale bars, 200 μm.

(G) Distance travelled in center, time spent in center area, central entries, total distance travelled, and percentage of distance in center (%) in the OFT of above representative mice with incorrect injection positions.

(H) Open arm distance travelled, open arm time spent, open arm entries, percentage of open arm entries, and closed arm entries in the EPM of above representative mice with incorrect injection positions.

**Fig. S3. Inhibition of CRFR2 in the PVT** **promotes explore behavior of mice in the central area of OFT at room temperature.**

(**A-E**) Open field test: distance travelled in center, time spent in center area, central entries, total distance travelled, and percentage of distance in center (%) (n = 6 per group). Representative trajectory plots depict the behavioral patterns of a single representative subject.

(F-J) Elevated plus-maze test: open arm distance travelled, open arm time spent, open arm entries, percentage of open arm entries, and closed arm entries (n = f per group). Representative trajectory plots represent the behavior of a single representative subject. Data presented as mean ± SEM, *P* value determined using unpaired *t* tests (**a-f**). **P* < 0.05.

**Fig. S4. The knock-down of CRFR2 in PVT decreases anxiety-like behavior in mice at room temperature.**

(A) Expression of sgRNA (NC) or sgRNA (CRFR2) virus in PVT neurons of Cas9 mice.

(B-C) Number of PVT c-Fos and CRFR2 positive neurons (green) in Cas9 mice (n = 3 per group). Scale bars, 200 μm.

(D-H) Open field test: distance travelled in center, time spent in center area, central entries, total distance travelled, and percentage of distance in center (%) (n = 8 per group). Representative trajectory plots depict the behavioral patterns of a single representative subject.

(I-M) Elevated plus-maze test: open arm distance travelled, open arm time spent, open arm entries, percentage of open arm entries, and closed arm entries (n = 8-9 per group). Representative trajectory plots represent the behavior of a single representative subject. Data presented as mean ± SEM, *P* value determined using unpaired *t* tests (B-M). **P* < 0.05.

**Fig. S5. Inhibition of Vglut2 excitatory neurons in PVT does not affect anxiety-like behavior in mice at room temperature.**

(A) Expression of hM4D (Gi) virus in Vglut2 neurons.

(B-F) Open field test: distance travelled in center, time spent in center area, central entries, total distance travelled, and percentage of distance in center (%) (n = 5-6 per group). Representative trajectory plots depict the behavioral patterns of a single representative subject.

(G-K) Elevated plus-maze test: open arm distance travelled, open arm time spent, open arm entries, percentage of open arm entries, and closed arm entries (n = 6 per group). Representative trajectory plots represent the behavior of a single representative subject. Data presented as mean ± SEM, *P* value determined using unpaired *t* tests (B-K).
